# Optimizing the Femoral Offset for Restoring Physiological Hip Muscle Function in Patients With Total Hip Arthroplasty

**DOI:** 10.3389/fbioe.2021.645019

**Published:** 2021-03-30

**Authors:** Xiangjun Hu, Nan Zheng, Yunsu Chen, Kerong Dai, Dimitris Dimitriou, Huiwu Li, Tsung-Yuan Tsai

**Affiliations:** ^1^School of Biomedical Engineering, Med-X Research Institute, Shanghai Jiao Tong University, Shanghai, China; ^2^Engineering Research Center of Digital Medicine and Clinical Translation, Ministry of Education, Shanghai, China; ^3^Shanghai Key Laboratory of Orthopaedic Implants, Clinical Translation R&D Center of 3D Printing Technology, Department of Orthopaedic Surgery, Shanghai Ninth People’s Hospital, Shanghai Jiao Tong University School of Medicine, Shanghai, China; ^4^Department of Orthopedic Surgery, Shanghai Jiao Tong University Affiliated Sixth People’s Hospital, Shanghai, China; ^5^Department of Orthopaedics, Bürgerspital Solothurn, Solothurn, Switzerland

**Keywords:** total hip arthroplasty, biomechanics, femoral offset, moment arm, hip muscles

## Abstract

**Objective:**

Femoral offset (FO) restoration is significantly correlated with functional recovery following total hip arthroplasty (THA). Accurately assessing the effects of FO changes on hip muscles following THA would help improve function and optimize functional outcomes. The present study aimed to (1) identify the impact of FO side difference on the hip muscle moment arms following unilateral THA during gait and (2) propose the optimal FO for a physiological hip muscle function.

**Methods:**

*In vivo* hip kinematics from eighteen unilateral THA patients during gait were measured with a dual-fluoroscopic imaging system. The moment arms of thirteen hip muscles were calculated using CT-based 3D musculoskeletal models with the hip muscles’ lines of actions. The correlation coefficient (R) between FO and hip muscle moment arm changes compared with the non-implanted hip was calculated. We considered that the FO reconstruction was satisfactory when the abductor moment arms increased, while the extensor, adductor, and flexor moment arms decreased less than 5%.

**Results:**

A decreased FO following THA was significantly correlated with a decrease of the abductor and external rotator moment arms during the whole gait (*R* > 0.5) and a decrease of extensor moment arms during the stance phase (*R* > 0.4). An increased FO following THA was significantly associated with shorter flexor moment arms throughout the gait (*R* < −0.5) and shorter adductor moment arms in the stance phase (*R* < −0.4). An increase in FO of 2.3–2.9 mm resulted in increased abductor moment arms while maintaining the maximum decrease of the hip muscles at less than 5.0%.

**Conclusion:**

An increase of 2–3 mm in FO could improve the abductor and external rotator function following a THA. Accurate surgical planning with optimal FO reconstruction is essential to restoring normal hip muscle function in THA patients.

## Introduction

Total hip arthroplasty (THA) is an effective method for treating end-stage hip diseases (Schmalzried et al., 2000; Michaelsson, 2014; Yi et al., 2019). Accurate biomechanical reconstruction of the hip anatomy following THA allows for physiological muscle activities and restoration of hip function with fewer complications (Girard et al., 2006; Rudiger et al., 2017). The femoral offset (FO) refers to the horizontal distance from the center of rotation of the femoral head to the femur’s long axis (Girard et al., 2006). Several studies have recognized FO’s importance in the soft-tissue tension, resultant force across the hip, and biomechanical recovery around the hip following THA (Sakalkale et al., 2001; Malviya et al., 2010; Liebs et al., 2014). Lack of FO restoration could lead to muscle imbalance, gait instability, increased polyethylene wear, and prosthetic joint dislocations (Dorr et al., 1983; Kiyama et al., 2010; Renkawitz et al., 2016).

Numerous studies reported that the decline of hip muscle function following THA was related to abnormal FO. A prospective cohort study of 222 patients demonstrated that a decrease in FO of more than 5 mm compared to the non-implanted side was associated with poor functional recovery, weak hip abductor muscle, and more use of the walking aids (Mahmood et al., 2016). Bjordal and Bjorgul (2015) found that intraoperative FO repair could promote the recovery of the abductor moment arms, thus rebuilding the balance of the peri-hip muscle group, which is conducive to the rehabilitation of joint function following THA. Excessive FO might increase polyethylene wear, resulting in hip muscle pain and function reduction (Little et al., 2009; Shapira et al., 2020b). Therefore, optimizing the FO for restoring physiological hip muscle function in patients with THA is crucial. However, up to date, there is no consensus regarding the optimal FO following THA. Previous studies primarily measured FO and abductor moment arms using plain radiographs and compared these measurements in static positions (Pierchon et al., 1994; Mahmood et al., 2016).

Femoral offset is associated with muscle function during daily activities. Sariali et al. (2014) reported that FO decreased by more than 15% induced gait disturbances. Rasch et al. (2010) found that hip muscles remained 6% weaker than the non-implanted side 2 years following THA. Asymmetric gait could aggravate muscle imbalance and increase the risk of dislocation, resulting in a vicious circle (Bahl et al., 2018). Muscle imbalance could further alter the gait pattern (Kolk et al., 2014). However, there is limited quantitative data on the association between the changes of FO and hip muscle performance during daily activity following THA. A previous study reported that the cause of hip pain and low-back pain during daily activities following THA is associated with the malfunction of deep external rotator muscles (Hernando et al., 2015). The contact between the iliopsoas tendon and the acetabular prosthesis under motion could also induce groin pain following THA (Park et al., 2016). Precise evaluation of the FO’s effects on the hip muscles in THA patients during gait could help improve functional outcomes and optimize the rehabilitation training program. There is limited quantitative data on the association between the changes of FO and the hip muscle moment arms through three-dimensional (3D) *in vivo* and *in vitro* measurement during gait after THA.

The purposes of the present study were to (1) identify the effect of FO side difference on the hip muscle moment arms following unilateral THA during gait and (2) propose the optimal FO, which would be the most beneficial for the recovery of physiological hip muscle function by restoring muscle moment arms following THA. This study adopted combined 3D computed tomography (CT)-based computer modeling and dual-fluoroscopic imaging system based on tracking technology to measure *in vivo* six-degrees-of-freedom (6-DOF) hip kinematics during gait.

## Materials and Methods

### Patients Demographics

The institutional Internal Review Board approved this study (No. 2019026). Written consent was obtained from each participant before taking part in this study project. Eighteen patients (13 women, 5 men; age 60.6 ± 9.0 years) with a good functional unilateral THA (Harris hip score >90 points) were enrolled. The average follow-up period was 10.4 ± 4.9 months. All subjects underwent a THA for hip osteoarthritis. Patients with previous surgical treatment or history of THA dislocation, subluxation, or periprosthetic fractures were excluded. THA was performed using the posterolateral approach.

### CT-Based 3D Modeling and Measurements of the Hip Kinematics During Gait

**FIGURE 1:**
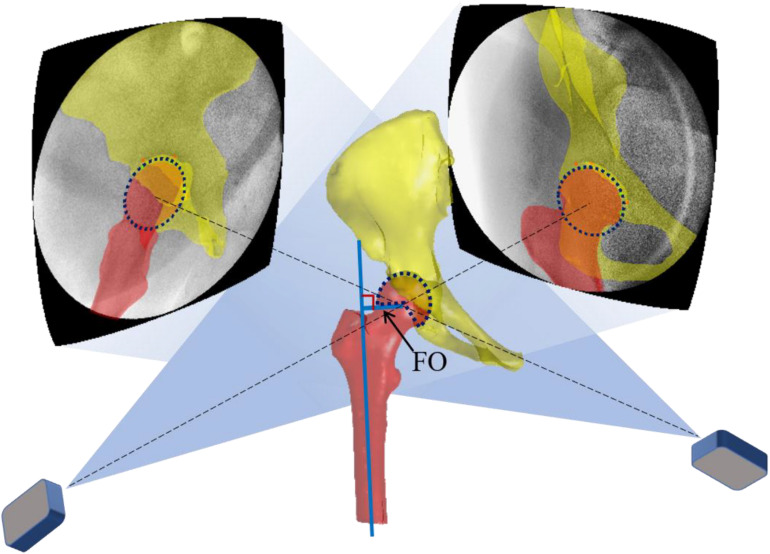
A virtual DFIS environment is constructed following the projection parameters obtained from the calibration procedure. The two-projection line intersection from two X-ray sources to the hip joint center determines the 3D skeletal models’ position. The FO was defined as the perpendicular distance from the femoral head’s rotation center to the femoral long axis.

All subjects followed the same study protocol using a CT (SOMATOM Definition AS1; Siemens, Germany) scan in the supine position, from the L5 vertebra to the tibial plateau with settings of 120 kV and 80 mA for the creation of 3D surface models of the femur, acetabular cup, pelvic, and prosthesis. The surface models’ outlines were reconstructed using a Gaussian filter with a gradient threshold and region growing, which were segmented and reconstructed (Amira, Thermo Fisher Scientific, Waltham, MA, United States). Then, each participant’s hip was simultaneously imaged using two fluoroscopes (BV Pulsera, Phillips Medical, United States) under snapshots (with an 8-ms pulse width, 60–80 kV, and 0.042–0.066 mAs) while walking on a treadmill at a self-selected speed. The same walking speed was set when testing the implanted and non-implanted hips of the same patient. The 3D surface models were then imported into a customized program in MATLAB (R2020a; MathWorks, Natick, MA, United States). The pelvic and femoral local coordinate system was defined using the bony landmarks on the 3D surface models, following the International Society of Biomechanics (ISB) recommendation (Wu et al., 2002). The processing procedure has been described in a previous publication (Tsai et al., 2014). Next, the fluoroscopic images, 3D surface models, and local coordinate systems were imported into MATLAB to simulate the laboratory’s real environment (Figure 1). The 3D joint models could be translated and rotated through 6-DOF in the 3D space in the virtual environment. For the hip translations, the 3D vector from the center of the acetabulum to the center of the femoral head in the acetabular coordinate system was measured. The tracking error for this technique is less than 0.35 mm and 0.55° in calculating hip joint translations and rotations (Tsai et al., 2013; Hu et al., 2020).

### Muscle Moment Arm

**FIGURE 2:**
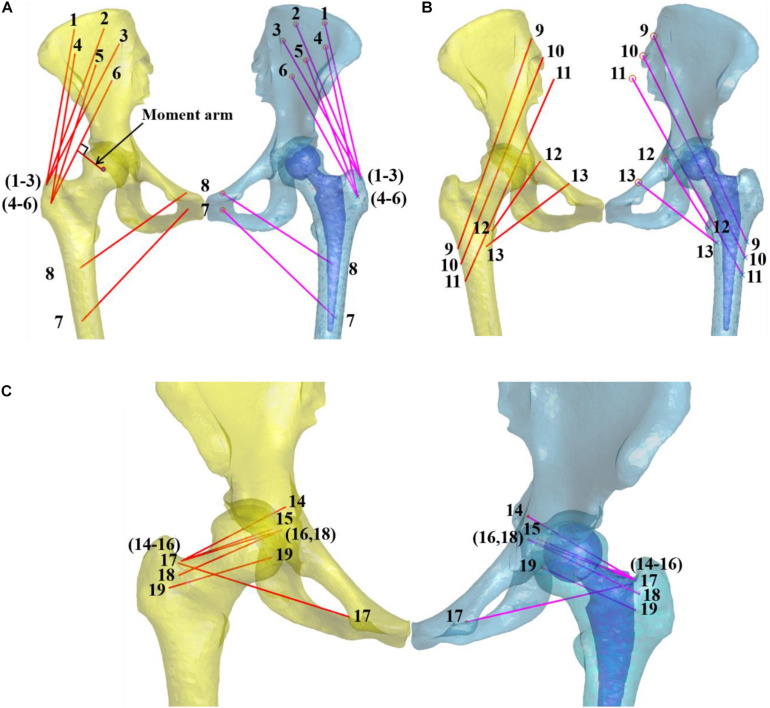
The pelvic and femoral bone models with the skeletal hip muscle lines of action. **(A)** The abductor and adductor include gluteus medius [anterior bundle (1, GMDA), median bundle (2, GMDM), posterior bundle (3, GMDP)], gluteus minimus [anterior bundle (4, GMIA), median bundle (5, GMIM), posterior bundle (6, GMIP)], adductor brevis (7, AB), and pectineus (8, PT). **(B)** The extensor muscle group include the gluteus maximus [anterior bundles (9, GMXA), median bundles (10, GMXM), posterior bundles (11, GMXP)]. The flexor muscle group includes the iliacus (12) and psoas (13). **(C)** The external rotation muscle group includes the gemellus superior (14, GS), gemellus inferior (15, GI), obturator internus (16, OI), obturator externus (17, OE), piriformis (18, PF), and quadratus femoris (19, QF). The muscle moment arm was defined as the femoral head’s rotation center perpendicular distance to each muscle simulation line. The moment arm of GMIP was shown at the arrow.

The 3D hip skeleton models were imported into MATLAB to mark the starting and ending attachment area of the hip muscles in the pelvis and femur of the non-implanted side according to the anatomy (Brand et al., 1982; Neumann, 2010) (Figure 2). Several muscles were divided according to their functions: the abductor muscle group includes gluteus medius (GMD) and gluteus minimus (GMI). The adductor muscle group includes adductor brevis (AB) and pectineus (PT). The external rotation muscle group includes gemellus superior (GS), gemellus inferior (GI), obturator internus (OI), obturator externus (OE), piriformis (PF), and quadratus femoris (QF). The flexor muscle group includes iliacus and psoas. The extensor muscle group includes gluteus maximus (GMX).

The muscle attachment areas’ centers were taken as starting and ending points to simulate the muscle tension lines (Dostal and Andrews, 1981). When the longitudinal direction of muscle bundles was a curve, the turning point of bone structure was considered the ending point (Brand et al., 1982; Hu et al., 2020). When the muscle attachment point was wide, it was divided into bundles to illustrate the respective functions of different muscle bundles, for example, gluteus maximus [anterior bundles (GMXA), median bundles (GMXM), posterior bundles (GMXP)], gluteus medius [anterior bundles (GMDA), median bundles (GMDM), posterior bundles (GMDP)], gluteus minimus [anterior bundles (GMIA), median bundles (GMIM), posterior bundles (GMIP)]. The hip muscles’ attachment sites were compared with existing meticulous anatomical studies to ensure consistency (Brand et al., 1982). The point to surface registration technique was used to align with the mirrored femur, and the remaining femur of the implanted side simulates the lines of action of each muscle of the implanted side (Tsai et al., 2014, 2015).

During gait, the spherical center of the femoral head was fitted to represent the center of rotation. The moment arm was defined as the perpendicular distance from the femoral head’s rotation center to each muscle’s line of action (Figure 2). The muscle moment arm difference between the implanted and non-implanted hip during gait was measured using the *in vivo* 6-DOF hip joint kinematics.

### Correlation Between FO Difference and Muscle Moment Arm Difference

The FO was defined as the perpendicular distance from the femoral head’s rotation center to the femoral long axis (Figure 1). The correlation between the hip muscle moment arm difference and FO difference between sides during gait was calculated. The correlation for each muscle varied during gait. The moment arm difference of each muscle was normalized relative to the moment arm of the non-replacement side. Then, a linear regression was used to analyze the relationship between moment arm difference and FO difference. The FO differences of 5 and 8 mm were widely considered in clinical practice and previous studies (Sariali et al., 2014; Mahmood et al., 2016; Shapira et al., 2020a). Thus, each muscle moment arm difference was calculated at the FO difference of 8, 5, 0, −5, and −8 mm on the linear regression lines. Arnold et al. (2000) reported that muscle moment arm changes less than 5% are desirable. To optimize the range of FO difference, we considered that the FO reconstruction was satisfactory when the abductor moment arm increased, while other muscle moment arm decreased less than 5% (Arnold et al., 2000). Thus, the optimal FO could be determined according to the calculated linear regression between the changes of the FO and muscle moment arms.

### Statistical Analysis

All measured parameters were expressed as an average and standard deviation. Pearson’s correlation was used to analyze the relationship between linear variables. A mathematical model of simple linear regressions was established for determining the effects of the FO difference on the hip muscle moment arm. The level of significance was defined as *p* < 0.05. Statistical analysis was performed using MATLAB, whereas sample size analysis was computed in G^∗^power (Franz Faul, Christian-Albrechts-Universität, Kiel, Germany).

## Results

### FO Value

The average FO of the non-implanted and implanted sides was 38.9 ± 3.7 mm and 39.9 ± 6.0 mm, respectively (*p* > 0.05) (Table 1).

**TABLE 1:** The average (AVG) and standard deviation (STD) of FO in THA and contralateral non-operated hip were illustrated during gait.

Patients	1	2	3	4	5	6	7	8	9	10	11	12	13	14	15	16	17	18	AVG	STD
NFO (mm)	37.8	46.7	44.1	25.5	44.9	37.6	36.9	45.4	37.1	40.7	44.5	45.9	40.6	28.5	46.2	41.9	38.2	35.5	39.9	6.0
TFO (mm)	36.9	41.2	45.0	33.0	42.3	37.3	37.0	39.2	34.8	32.2	44.8	42.7	36.7	37.8	41.9	40.1	38.6	38.5	38.9	3.7
DFO (mm)	–0.9	–5.5	0.9	7.5	–2.6	–0.2	0.1	–6.2	–2.3	–8.5	0.3	–3.2	–3.9	9.2	–4.4	–1.7	0.4	2.9	–1.0	4.4

*NFO referred to native FO, TFO referred to THA FO, DFO referred to the difference value between NFO and TFO.*

### Correlation Between FO Difference and Hip Muscle Moment Arm Difference

**FIGURE 3:**
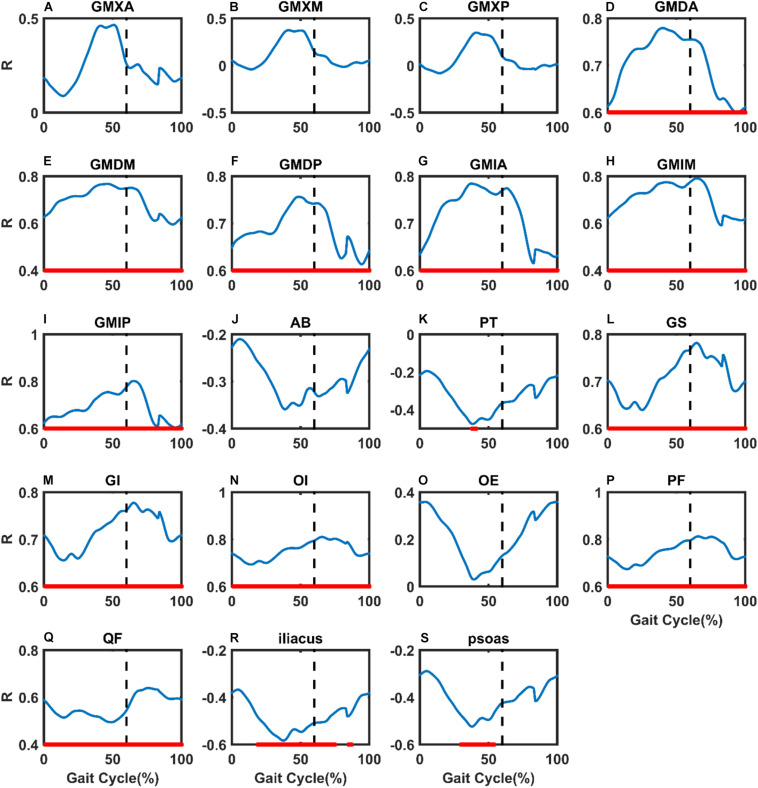
The changes in the correlation coefficient (R) between FO difference and hip muscle moment arm difference compared with non-implanted hip in THA patients were illustrated during gait. **(A)** Gluteus maximus anterior bundle (GMXA), **(B)** gluteus maximus median bundle (GMXM), **(C)** gluteus maximus posterior bundle (GMXP), **(D)** gluteus medius anterior bundle (GMDA), **(E)** gluteus medius median bundle (GMDM), **(F)** gluteus medius posterior bundle (GMDP), **(G)** gluteus minimus anterior bundle (GMIA), **(H)** gluteus minimus median bundle (GMIM), **(I)** gluteus minimus posterior bundle (GMIP), **(J)** adductor brevis (AB), **(K)** pectineus (PT), **(L)** gemellus superior (GS), **(M)** gemellus inferior (GI), **(N)** obturator internus (OI), **(O)** obturator externus (OE), **(P)** piriformis (PF), **(Q)** quadratus femoris (QF), **(R)** iliacus, and **(S)** psoas. Nearly 60% of the black vertical dashed lines indicate toe-off. Areas of statistical significance were represented by the bold red line along the *X*-axis of each graph. *X*-axis: percentage of the gait cycle, *Y*-axis: R values.

Highly positive correlations were found throughout the gait cycle in GMD, GMI, GS, GI, OI, PF, and QF (Figures 3D–I,L–N,P,Q). The maximum correlation coefficients reached 0.78, 0.80, 0.78, 0.78, 0.81, 0.81, and 0.64 (*p* = 0.0001, 0.0000, 0.0001, 0.0001, 0.0000, 0.0000, and 0.004, respectively). During the single-leg stance phase to double-leg stance phase gait cycle in GMXA (Figure 3A), a positive correlation between FO difference and muscle moment arm difference was observed, in which the maximum correlation coefficient reaches 0.47 (*p* = 0.05). Besides, during the early support phase and terminal swing phase of the gait cycle in OE, a positive correlation was also found (Figure 3O), in which the maximum correlation coefficient reaches 0.36 (*p* = 0.14). On the contrary, a negative correlation during the stance phase gait cycle was found in AB and PT (Figures 3J,K), in which the maximum correlation coefficient reached −0.36 and −0.48 (*p* = 0.14, 0.05). A highly negative correlation was measured in iliacus and psoas during nearly the whole gait cycle (Figures 3R,S), in which the maximum correlation coefficient reached −0.58 and −0.52 (*p* = 0.01, 0.03).

**FIGURE 4:**
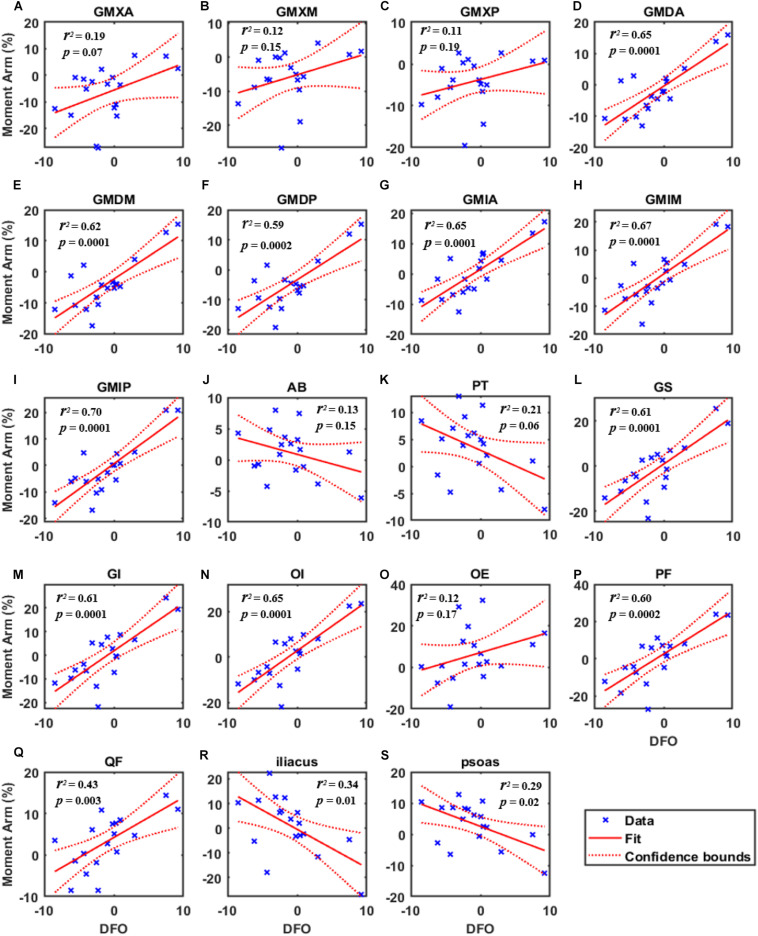
The simple linear regression curve showed the highest correlations of the moment arm change ratio [Moment Arm (%)] with the FO difference (DFO) during gait. **(A)** Gluteus maximus anterior bundle (GMXA), **(B)** gluteus maximus median bundle (GMXM), **(C)** gluteus maximus posterior bundle (GMXP), **(D)** gluteus medius anterior bundle (GMDA), **(E)** gluteus medius median bundle (GMDM), **(F)** gluteus medius posterior bundle (GMDP), **(G)** gluteus minimus anterior bundle (GMIA), **(H)** gluteus minimus median bundle (GMIM), **(I)** gluteus minimus posterior bundle (GMIP), **(J)** adductor brevis (AB), **(K)** pectineus (PT), **(L)** gemellus superior (GS), **(M)** gemellus inferior (GI), **(N)** obturator internus (OI), **(O)** obturator externus (OE), **(P)** piriformis (PF), **(Q)** quadratus femoris (QF), **(R)** (iliacus), and **(S)** psoas. The saltire represented the data of one subject during gait. *p*: significant level, *r*^2^: coefficient of determination.

Pearson correlation coefficient analysis in each muscle revealed a significant correlation between the moment arm difference and FO difference relative to the non-implanted sides (Figure 4). When the FO decreased by 5.0 mm, the moment arms of GMXA, GMDP, GMIP, and GS decreased by 10.6, 10.8, 8.9, and 9.7%, respectively (Table 2). When the FO reduced by 8 mm, the moment arms of PF, GS, and GI reduced by 16.1, 16.0, and 14.3%, respectively (Table 2). When the FO in the non-implanted side and the implanted side were equal, the moment arms of GMXA, GMXD, and GMXP decreased by 5.6, 5.2, and 3.7%, respectively (Table 2). FO increased by 8 mm, and the muscle with the greatest reduction in the moment arm was the iliacus, reaching 12.9% (Table 2). When the FO increased by 2.3–2.9 mm, the abductor moment arms increased while all muscle moment arms decreased by less than 5.0% (Tables 2, 3).

**TABLE 2:** The normalized muscle moment arm difference of extensor, abductor, adductor, external rotator and flexor between both sides at different FO differences.

Moment arm difference (%)																			

DFO	Extensor			Abductor						Adductor		External rotator						Flexor	
					
	GMX			GMD			GMI			AB	PT	GS	GI	OI	OE	PF	QF	Iliacus	Psoas
			
	GMXA	GMXM	GMXP	GMDA	GMDM	GMDP	GMIA	GMIM	GMIP										
8 mm	2.4	–0.2	–0.2	11.3	9.5	8.4	13.1	15.2	15.9	–1.6	–1.6	17.8	17.9	20.0	15.0	21.2	11.9	–12.9	–4.2
5 mm	–0.6	–2.1	–1.5	6.9	5.0	4.0	8.8	10.1	10.2	–0.7	0.1	11.4	11.9	13.6	12.0	14.2	9.0	–8.3	–1.7
0 mm	–5.6	–5.2	–3.7	–0.4	–2.3	–3.4	1.5	1.5	0.6	0.9	3.0	0.9	1.8	2.8	7.1	2.6	4.2	–0.5	2.5
−5 mm	–10.6	–8.3	–5.9	–7.7	–9.7	–10.8	–5.8	–7.0	–8.9	2.4	5.9	–9.7	–8.2	–8.0	2.2	–9.1	–0.6	7.3	6.7
−8 mm	–13.6	–10.2	–7.2	–12.1	–14.1	–15.2	–10.1	–12.1	–14.6	3.3	7.6	–16.0	–14.3	–14.4	–0.8	–16.1	–3.5	12.0	9.2

*When the FO difference was 8 mm, 5 mm, 0 mm, −5 mm, and −8 mm, each muscle moment arm difference was listed.*

**TABLE 3:** The FO difference based on the linear regression curve at different normalized muscle moment arm differences of extensor, abductor, adductor, external rotator and flexor.

DFO (mm)																			

Moment arm difference	Extensor			Abductor						Adductor		External rotator						Flexor	
					
	GMX			GMD			GMI			AB	PT	GS	GI	OI	OE	PF	QF	iliacus	psoas
			
	GMXA	GMXM	GMXP	GMDA	GMDM	GMDP	GMIA	GMIM	GMIP										
−5%	0.6	0.3	–3.0	–3.1	–1.8	–1.1	–4.5	–3.8	–3.0	19.1	13.9	–2.8	–3.4	–3.6	–12.3	–3.2	–9.5	2.9	9.0
0	5.6	8.3	8.5	0.3	1.6	2.3	–1.0	–0.9	–0.3	2.8	5.2	–0.4	–0.9	–1.3	–7.2	–1.1	–4.4	–0.3	3.0
5%	10.5	16.4	19.9	3.7	5.0	5.7	2.4	2.0	2.3	–13.5	–3.5	1.9	1.6	1.0	–2.1	1.0	0.8	–3.5	–3.0

*When the hip muscles moment arms difference was −5%, 0%, and 5%, the FO difference was listed.*

## Discussion

Statistically significant correlations between FO difference and the hip muscle moment arm difference compared with the non-implanted side were found in unilateral THA patients during gait. Our study revealed that a decreased FO was significantly correlated with decreased abductor and external rotator moment arms throughout the gait (*R* > 0.5, *P* < 0.01, Tables 2, 3 and Figures 3, 4) and decreased extensor moment arms during the stance phase in THA (*R* > 0.4, *P* > 0.05, Tables 2, 3 and Figures 3, 4). Besides, an increase in FO was significantly correlated with decreased flexor moment arms during almost all gait (*R* < −0.5, *P* < 0.05, Tables 2, 3 and Figures 3, 4) and decreased adductor moment arms during the stance phase of the gait (*R* < −0.4, *P* < 0.05, Tables 2, 3 and Figures 3, 4). A decrease in FO demonstrated more significance on hip muscle moment arms than an FO increase. An increase in FO (by approximately 2–3 mm) during THA could improve abductor, external rotator moment arms.

Numerous studies have shown that a reduction of abductor moment arm following THA is related to an FO decrease. Bjordal and Bjorgul (2015) found that FO was positively correlated with the abductor muscle moment arm following THA. Intraoperative FO restoration could recover the abductor muscle moment arm, thus rebuilding the balance of the peri-hip muscle group, which is conducive to the rehabilitation of joint function after THA (Bjordal and Bjorgul, 2015). McGrory et al. (1995) performed hip X-ray, range of motion (ROM), and abductor muscle strength measurements in 64 patients who underwent THA 1 year after surgery. The results showed that FO was positively correlated with ROM in a linear regression analysis (*p* = 0.046; *r* = 0.22), the abductor muscle strength (*p* = 0.0001; *r* = 0.40), and the abduction moment arm (*p* = 0.0001; *r* = 0.43) (McGrory et al., 1995). In a dynamical musculoskeletal OpenSim model of 15 THA hips, Rudiger et al. (2017) quantitatively described the effect of FO changes on abductor moment arms during gait, which demonstrated that with the decrease of FO reconstructions, muscle moment arms decreased. The findings are consistent with the results of our study.

The present study demonstrated that the FO difference affects also the other hip muscles, such as the external rotators. The external rotator muscles are located in the deep layer of the gluteus maximus, known as the deep external rotator muscles, and play the role of stabilizing the pelvis (Neumann, 2010). The direction of the muscle tension line and FO direction tend to be parallel. When FO increases, the spatial position of the ending point changed, the muscle tension line is far from the femoral rotation center, and the muscle moment arms increase accordingly. The length of the external rotator muscles is shorter, and the position near the femoral head is more susceptible to FO’s change. In the present study, a decrease in FO was significantly correlated with decreased external rotator moment arms (Table 2). A decrease in external rotator moment arm following THA may explain the excessive internal rotation during gait reported in THA patients (Tsai et al., 2015). A previous study reported that the deep external rotator muscle was implicated as the pathological source of hip pain and low-back pain following THA (Hernando et al., 2015). These complications might associate with the adverse biomechanical effects caused by the loss of FO.

Negative correlations were found between the flexor muscle moment arm changes and FO difference following THA. The iliacus and psoas in flexor muscles are collectively called iliopsoas muscle (Neumann, 2010). Our findings indicated that a decreased FO was significantly correlated with a decrease in the iliacus moment arm (Table 2). When the FO increases, the femur moves laterally, the tension line of the muscles is closer to the center of femoral rotation, and the moment arm decreases. Iliopsoas tendon impingement with the acetabular prosthesis is one potential complication following THA (Park et al., 2016). It is speculated that FO increase might move the iliopsoas muscle closer to the acetabular prosthesis and therefore increase the risk of impingement. Besides, the effect of FO on muscle moment arms also depends on patient anatomy such as femoral anteversion. Therefore, blindly increasing FO would reduce the efficiency of flexors, resulting in muscle imbalance and complications.

Our study found that when the FO in the non-implanted side and the implanted side were equal, the GMX moment arms decreased (Table 2). It is suggested that restored femoral offset following THA might not demonstrate superior outcomes. Besides, when FO decreased by 5 mm, the moment arms of GMXA, GMDM, GMDP, and GS decreased by approximately 10% (Table 2). Rudiger et al. (2017) reported that a loss of 8% of abductor moment arms resulted in a 16% increase in their forces. However, when FO increased by 5 mm, the moment arms of GMX, AB, iliacus, and psoas decreased by less than 5% (Table 2). These findings suggest that an FO loss tends to exhibit a more significant influence than an FO increase, which agrees with a previous systematic review (Shapira et al., 2020b). According to the analysis of the linear regression model, when the FO increased by 2.3–2.9 mm, the abductor and external rotator moment arms increased, and the other muscle moment arms decreased by less than 5% (Tables 2, 3). Nankaku et al. (2016) have reported that an abnormal gait pattern caused by asymmetrical frontal motion was associated with muscle atrophy of the hip abductor muscle before THA. The hip abductor moment arm rising due to an FO increase following THA may compensate for abductor weakness caused by muscle atrophy, which could improve work efficiency and improve gait imbalance. Our data suggest that an appropriate increase of 2–3 mm in FO could improve abductor, external rotator function recovery and reduce the adverse effects of THA on the extensor, adductor, and flexor. As a consequence, an optimal FO may reduce muscle imbalance, gait instability, and THA dislocations.

Dorr et al. (1983) found that the long-term hip muscle imbalance will increase the bending moment of the femoral prosthesis, causing loosening and fracture of the prosthesis. If the effect of the whole operation is taken into account, the deviation of FO cannot be avoided. The stability of the hip can be improved by increasing individualized muscle strength training to compensate for the reduction of the moment arm caused by surgery and generate greater torque. When FO difference is less than the recommended range (2.3–2.9 mm), focus on the training extensor, abductor, and external rotator, while when FO is larger than the range, focus on the training adductor and flexor. Strengthen the control and coordination ability between muscle groups to improve operational effectiveness.

Several limitations of the study should be noted. There was a lack of preoperative data to clarify the effects of FO on hip muscle moment arms in THA patients. However, the current study provided valuable information by comparing the effects between the implanted side and the non-implanted side. Using the correlations between FO and hip muscle moment arm changes and non-implanted hips, calculated by G-Power software, the statistical verification power of the data is 96%. Longer follow-ups would be expected to assess the impact of different anatomical changes such as femoral anteversion and rehabilitation training on muscle function recovery.

## Conclusion

The present study quantified the effect of FO difference following unilateral THA on the *in vivo* dynamic hip muscle moment arms during gait. FO loss tended to result in a more significant influence than an FO increase. It indicated that an increase of 2–3 mm in FO could improve abductor and external rotator function recovery while reducing the adverse effects of THA on the extensor, adductor, and flexor. When FO difference is less than the recommended range (2.3–2.9 mm), the patients should focus on training the hip extensors, abductors, and external rotators. When the FO is larger than the optimal range, the focus should turn on training hip adductors and flexors. Optimizing the FO for restoring the hip muscle balance could improve hip muscle strength, reduce postoperative complications, and promote the functional reconstruction of the hip joint.

## Data Availability Statement

The raw data supporting the conclusions of this article will be made available by the authors, without undue reservation, to any qualified researcher.

## Ethics Statement

The studies involving human participants were reviewed and approved by the Ethics Committee of Shanghai Sixth People’s Hospital, China (No. 2019026). Written informed consent was obtained from all patients enrolled in the investigation. The patients/participants provided their written informed consent to participate in this study.

## Author Contributions

XH, NZ, YC, HL, and T-YT contributed to the conception and design. XH, NZ, YC, HL, and T-YT contributed to the provision of study materials or patients and collection and assembly of data. XH and T-YT carried out the data analysis. XH, NZ, HL, KD, and T-YT contributed to data interpretation. XH, NZ, DD, and T-YT participated in the writing of the manuscript. All authors gave approved the final manuscript.

## Conflict of Interest

The authors declare that the research was conducted in the absence of any commercial or financial relationships that could be construed as a potential conflict of interest.
